# Supplementary figures and images for: Efficient CRISPR/Cas9 genome editing in a salmonid fish cell line using a lentivirus delivery system
Source: BMC Biotechnol. 2020 Jun 23;20:35. doi: 10.1186/s12896-020-00626-x (PMC7310381; doi:10.1186/s12896-020-00626-x)

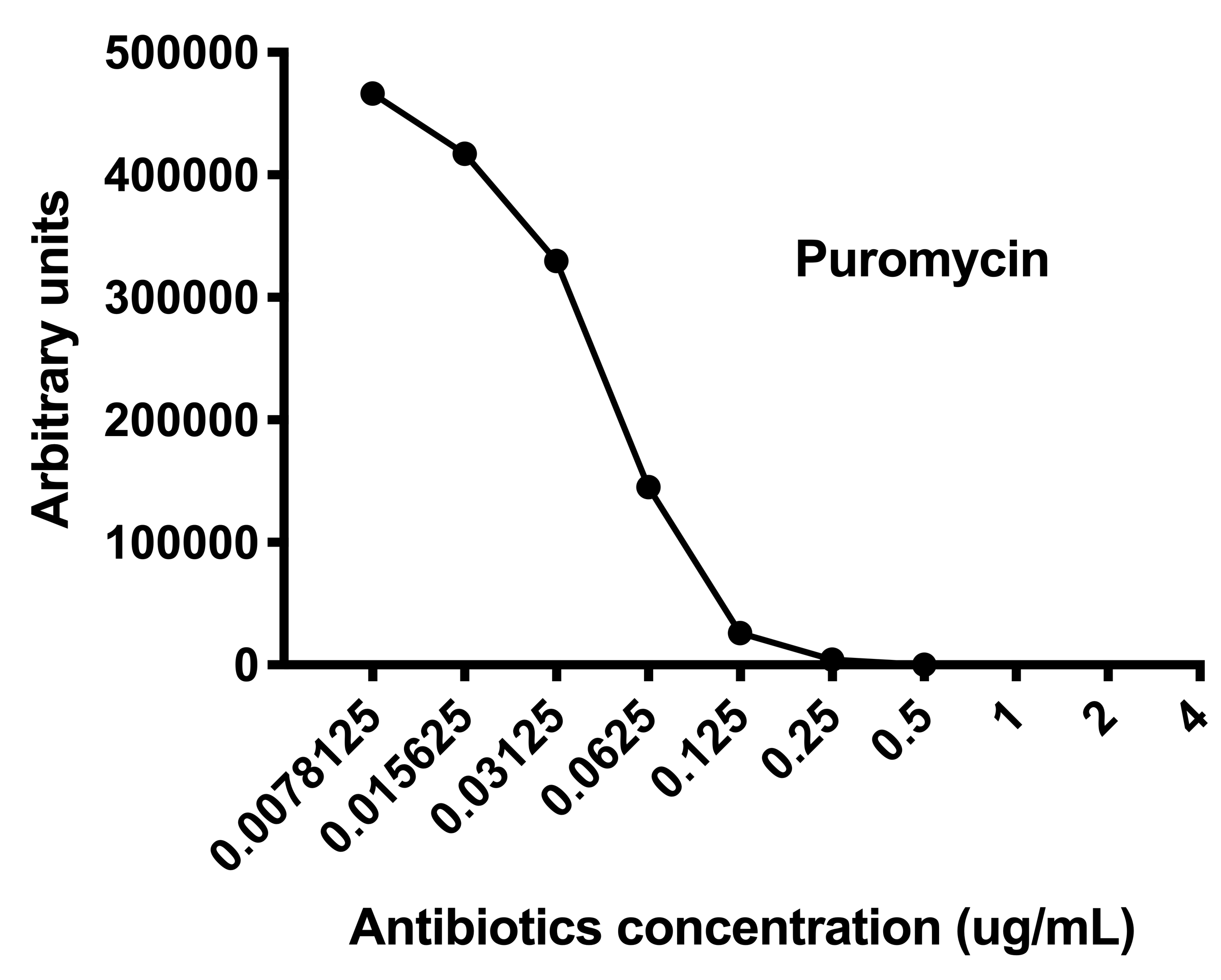

Supplement: Supplementary file 1 — Additional file 1 Figure S1. Puromycin can be used to select for resistant cells. CHSE-EC cells were treated for 7 days with different concentrations of Puromycin and the survival was calculated by CellTiter-Glo. A concentration of 0.25 μg/mL of puromycin was found to be the minimal concentration to efficiently kill all non-antibiotic-resistant cells. [file 12896_2020_626_MOESM1_ESM.png]

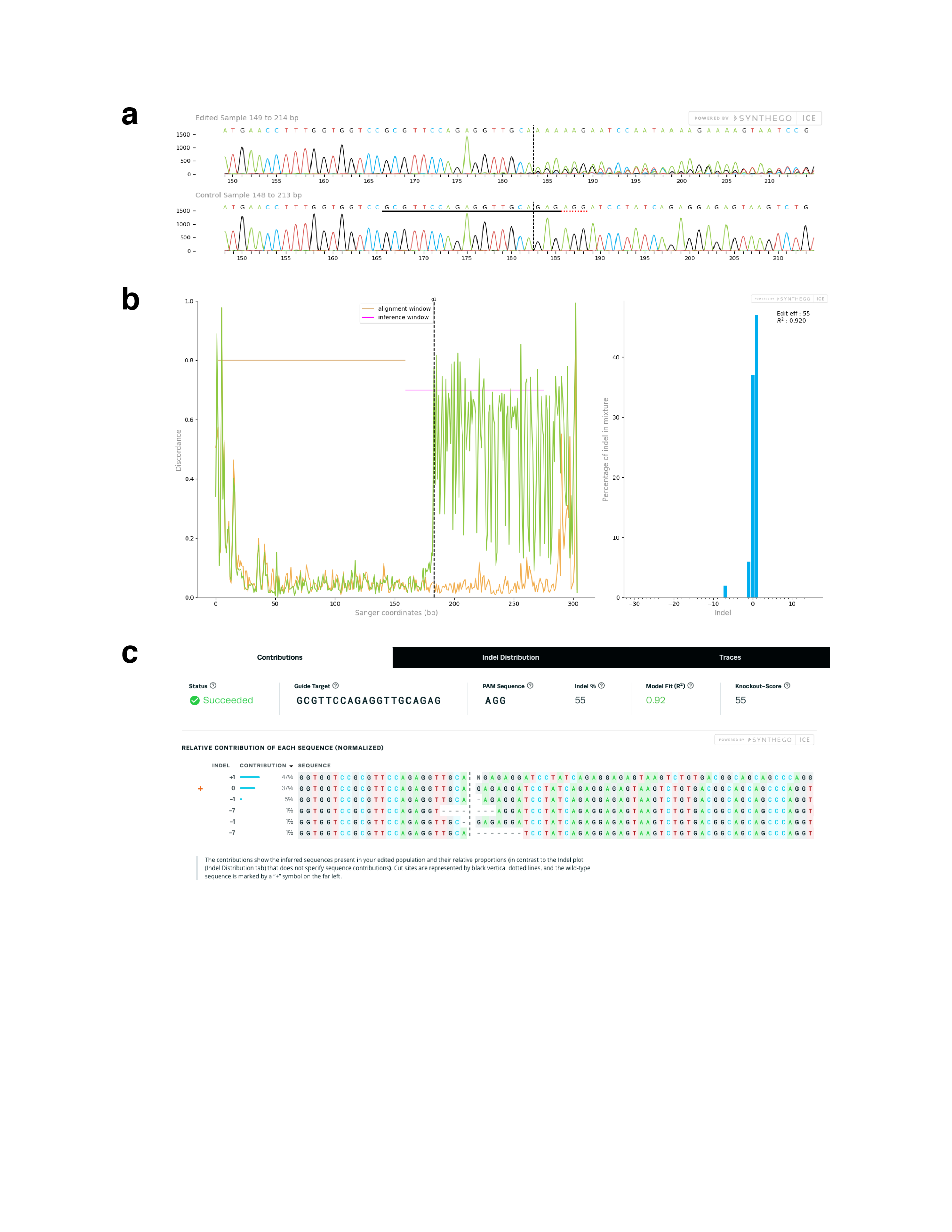

Supplement: Supplementary file 2 — Additional file 2 Figure S2. Editing efficiency estimation, The analysis of the editing of pooled cell population samples using ICE online software. a. The chromatograms (.ab1 file) from the control (non-edited) and edited samples, along with the gRNA sequence are uploaded on ice.sythego.com. b. The platform verifies that the cut site corresponds to the start of the mixed population chromatogram and deconvolutes the picks to original sequences + or – a few bases. c. The results are presented as the percentage of each edited sample present in the pooled population contributing to the mixed chromatogram. [file 12896_2020_626_MOESM2_ESM.png]

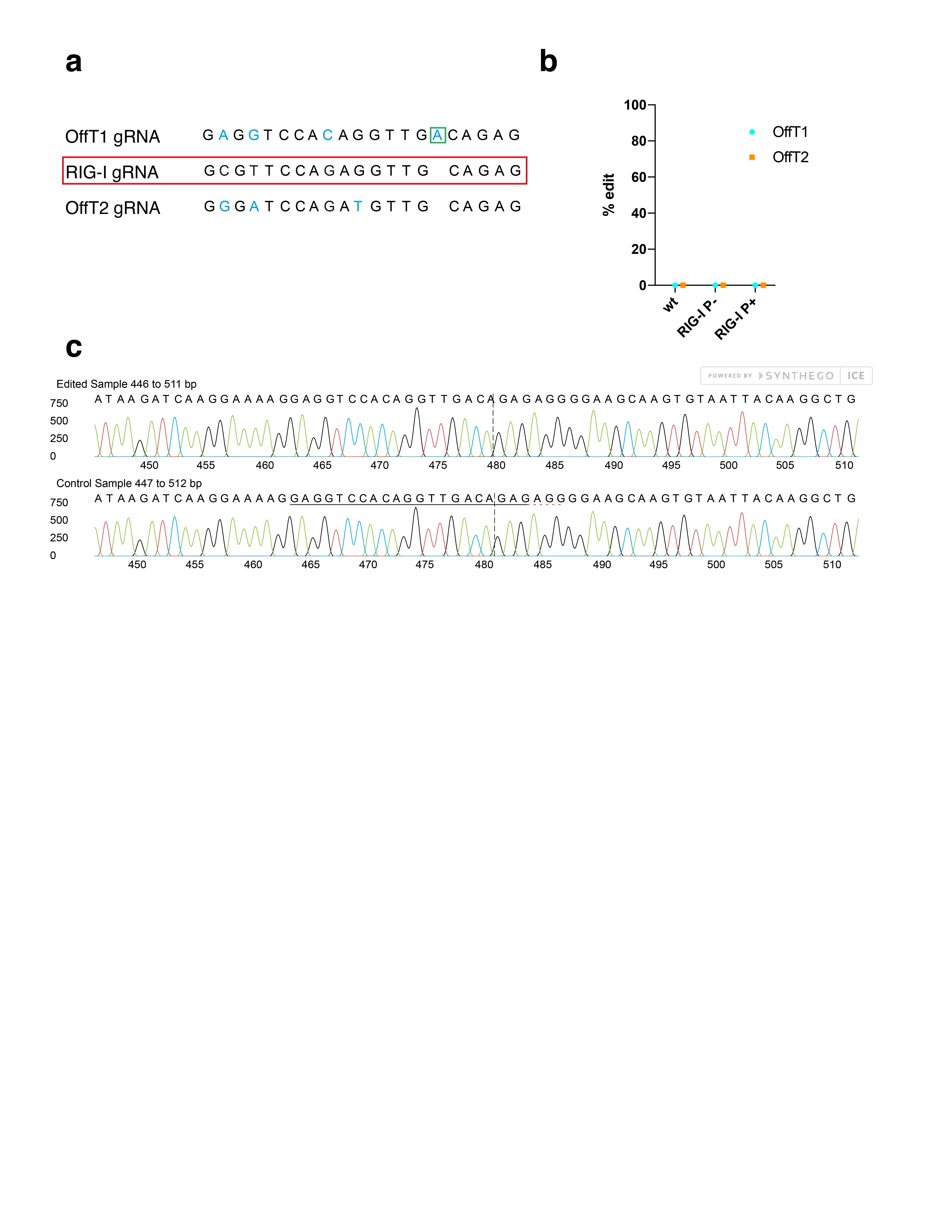

Supplement: Supplementary file 3 — Additional file 3 Figure S3. Off-target editing. Off-target evaluation of RIG-I editing. a: The sequence of the top 2 off-target sites are represented along the gRNA sequence used for targeting RIG-I (red box in the middle). Blue letters indicate differences with the original sequence. An additional nucleotide was sequenced in the CHSE-EC cell line, not present in the published sequence (Otsh_v1.0, green box). b: Diagram representing the editing efficiency in the off-target regions (OffT1: ch7:73101728–73,102,398 and OffT2: ch14:42341999–42,342,859). No off-target was detected by Sanger sequencing in either sample (Puro- and Puro+; all sequences, including CHSE-EC (wt) were compared to CHSEwt). c: Representative chromatogram from the sequencing of off-target region 1 (OffT1) in CHSEwt (Control sample, bottom track) and CHSE-EC-RIG-I Puro + (Edited Sample, top track). [file 12896_2020_626_MOESM3_ESM.png]
